# Influence of Complete Lesion Removal During Vacuum-Assisted Breast Biopsy on the Upgrade Rate of B3 Lesions Presenting as Microcalcifications

**DOI:** 10.3390/jcm14051513

**Published:** 2025-02-24

**Authors:** Giovanni Irmici, Catherine Depretto, Alessandra Pinto, Gianmarco Della Pepa, Elisa D’Ascoli, Claudia De Berardinis, Alice Bonanomi, Eleonora Ancona, Daniela Ballerini, Lidia Rabiolo, Simone Schiaffino, Andrea Cozzi, Gianfranco Scaperrotta

**Affiliations:** 1 Breast Radiology Department, Fondazione IRCCS Istituto Nazionale dei Tumori, Via Giacomo Venezian 1, 20133 Milan, Italy; 2 Postgraduation School in Radiodiagnostics, Università degli Studi di Pavia, Viale Camillo Golgi, 19, 27100 Pavia, Italy; 3 Radiology Unit, Diagnostic and Therapeutic Services, IRCCS ISMETT, Via Ernesto Tricomi 5, 90127 Palermo, Italy; 4 Imaging Institute of Southern Switzerland (IIMSI), Ente Ospedaliero Cantonale (EOC), Via Tesserete 46, 6900 Lugano, Switzerland; 5 Faculty of Biomedical Sciences, Università della Svizzera Italiana, Via Giuseppe Buffi 13, 6900 Lugano, Switzerland

**Keywords:** breast cancer, B3 lesions, vacuum-assisted breast biopsy, mammography, microcalcifications

## Abstract

**Background**: B3 lesions of the breast, for which vacuum-assisted biopsy (VABB) represents the standard tissue sampling approach, have different risks of upgrade to malignancy at surgery and/or follow-up. This study aimed to investigate if complete or partial lesion removal during VABB of B3 lesions presenting as microcalcifications influences their subsequent upgrade rate. **Methods**: For this retrospective single-center study, we retrieved 165 lesions diagnosed as B3 at VABB that presented solely as microcalcifications categorized as Breast Imaging Reporting & Data System (BI-RADS) 4 or 5 at mammography between January 2016 and December 2020. Surgical pathology or at least 3-year follow-up were obtained to determine potential lesion upgrade to malignancy. χ^2^, Fisher’s, and Mantel–Haenszel tests were performed to assess if complete lesion removal influenced upgrade rates overall and among different B3 subtypes. **Results**: Complete lesion removal was achieved in 99/165 cases (60.0%) and did not differ among B3 subtypes (*p* = 0.092). The overall upgrade rate was 8.5% (95% confidence interval [CI] 5.1–13.7%, 14/165), without statistically significant differences among B3 subtypes (*p* = 0.562). Conversely, completely removed lesions (4.0%, 95% CI 1.6–9.9%) had a statistically significant lower upgrade rate compared to partially removed lesions (15.2%, 95% CI 8.4–25.7%, *p* = 0.019). According to stratified analysis according to B3 subtypes, the odds ratio of upgrade among completely and partially removed flat epithelial atypia (0.13, 95% CI 0.00–1.45) was lower (Mantel-Haenszel test *p* = 0.016) than those of atypical ductal hyperplasia (0.31, 95% CI 0.02–3.17) and of lobular neoplasia (0.73, 95% CI 0.01–60.62). **Conclusions**: The upgrade rate of B3 lesions is significantly influenced by complete lesion removal, both overall and among different B3 subtypes.

## 1. Introduction

The investigation of breast disease is largely centered on breast cancer, which remains the leading cause of cancer-related mortality among women, accounting for approximately 140,000 deaths annually (16.4% of total female cancer deaths) in Europe [1]. Breast cancer is traditionally classified into two broad categories: invasive carcinoma and carcinoma in situ, which differ in terms of biological behavior, oncological risk, and management strategies [2]. Over the past decades, the European standardized incidence rate of in situ lesions has increased nearly fourfold, reaching 20.68 per 100,000 women in 2011 from 4.90 in 1989 [3] with ductal carcinoma in situ accounting for 15–25% of all breast cancer diagnoses [2]. Of note, despite the increasing detection of DCIS, its clinical significance remains debated, as DCIS is associated with a 10-year breast cancer specific survival of approximately 98% [3]: this discrepancy highlights the need for a more tailored approach to management based on individualized risk assessment.

Beyond in situ and invasive breast cancer, there is another category of breast lesions that does not meet the criteria for malignancy but still poses a major clinical dilemma: breast lesions of uncertain malignant potential (B3 lesions). The prevalence of these lesions varies between 3% and 21%, sometimes reaching considerable proportions in screening populations [4]. Managing B3 lesions remains challenging due to their heterogeneous nature, their different risks of upgrading to malignancy at surgical excision or during follow-up that ranges from 6 to 32% [5], and the need for careful risk stratification to guide clinical decision-making.

B3 lesions encompass a variety of subtypes: atypical ductal hyperplasia (ADH), flat epithelial atypia (FEA), lobular neoplasia (LN)—which includes lobular carcinoma in situ and atypical lobular hyperplasia—papillary lesions (PL), radial scars (RS), and other miscellaneous entities [6,7].

One of the most common breast imaging presentations of B3 lesions is the presence of suspicious microcalcifications at X-ray-based examinations (mammography, digital breast tomosynthesis or contrast-enhanced mammography) [4,8,9,10], which are increasingly prevalent due to the widespread implementation of digital mammography and digital breast tomosynthesis in population-based screening programmes [11,12].

Stereotactic vacuum-assisted breast biopsy (VABB) represents the standard approach for tissue sampling of lesions presenting solely as microcalcifications [13]. When used with large calibre needles, VABB allows for the collection of adequate and representative tissue samples, ensuring reliable histopathological assessment. Additionally, when technically feasible, VABB can achieve complete removal of the lesion, reducing the need for additional surgical procedures and supporting a more tailored approach to patient management [4,14,15].

In the context of therapeutic de-escalation [16], several studies have demonstrated that B3 lesions completely removed using stereotactic VABB may not require subsequent surgical intervention [17,18,19,20]. According to the guidelines of the Third International Consensus Conference, the distinction between complete and partial lesion removal is a key factor in the management of B3 lesions [6]. Specifically, for certain lesions such as FEA, LN, RS and PL, radiological follow-up may be deemed sufficient if the visible lesion has been almost or completely removed [6]. Also according to the German Gynecological Oncology Group guidelines, surgery can be avoided when microcalcifications are almost completely removed (>90%) in post-VABB imaging in patients with FEA [21].

However, while these findings highlight the potential of VABB in reducing unnecessary surgeries, a full understanding of its role in clinical practice is limited by a gap in the scientific literature concerning follow-up data for different B3 lesions presenting solely as microcalcifications. For these reasons, our study aims to evaluate whether complete or partial removal of B3 lesions presenting solely as microcalcifications with stereotactic VABB, could influence the overall upgrade rate—immediately at surgery and in a follow-up framework of 3 years—to malignancy, overall and among different B3 subtypes.

## 2. Materials and Methods

### 2.1. Study Design

This retrospective single-center study analyzed all consecutive stereotactic VABBs that were performed at a third-level cancer center (Fondazione IRCCS Istituto Nazionale dei Tumori, Milan, Italy) from January 2016 to December 2020 for suspicious microcalcifications detected at population-based or opportunistic screening mammography and categorized as Breast Imaging Reporting & Data System (BI-RADS) 4 or BI-RADS 5. For the purposes of this study, we included all B3 lesions diagnosed according to the “World Health Organization classification of tumors of the breast—2019” [7] that had an available final pathologic diagnosis at surgical excision or at least 36 months of imaging follow-up. B3 lesions presenting with other radiological features associated with microcalcifications, such as masses, architectural distortion and asymmetry, were excluded. The need for patients’ informed consent was waived due to the retrospective nature of the study.

### 2.2. Stereotactic VABB

Stereotactic VABBs were performed using 9G needles with the Eviva ATEC^®^ system (Hologic Inc., Marlborough, MA, USA) by different breast radiologists with more than 7 years of experience in stereotactic VABB. After thorough history taking, assessment of coagulation parameters, and acquisition of informed consent, the patient was positioned on the prone biopsy system (Affirm Prone Biopsy System, Hologic Inc., Marlborough, MA, USA). The breast with suspicious microcalcifications was compressed and digital breast tomosynthesis was performed to locate the target lesion. Once the lesion was localized, its spatial coordinates were sent to the biopsy system, and local anesthesia with 20 mg/mL lidocaine was administered, with the dose adjusted to the weight of the patient. To ensure that lidocaine did not cause displacement of microcalcifications, an additional mammogram was performed. After needle insertion, pre- and post-fire projections were taken to verify correct targeting and confirm that the needle was positioned in the correct location.

In each procedure, 12 or more specimens were taken per lesion, depending on different criteria (quality of specimens, target lesion size, patient compliance and correct needle position); at the end of each VABB procedure, lesions were classified as completely removed if all target microcalcifications had been successfully excised. Conversely, cases where residual microcalcifications were still present were categorized as having partial lesion removal. Regardless of the presence of residual microcalcifications, a non-magnetic marker was placed at the biopsy site in all cases. All procedures were followed by specimen mammography to confirm the presence of microcalcifications.

### 2.3. Lesion Management and Upgrade Definition

B3 lesions were classified according to the European guidelines for quality assurance in breast cancer screening and diagnosis [4]. Specifically, each lesion was categorized as ADH, FEA, LN, PL or RS.

The decision to refer cases to surgery or follow-up was made within a multidisciplinary meeting, taking into account the clinical characteristics, imaging findings, and pathology results of each patient. Surgical excision was performed after ultrasound or mammographic localization with a magnetic clip, typically within 4 weeks. Mammography of the surgical specimen was performed intraoperatively to ensure the complete inclusion of the area in the excised sample. In cases of incomplete resection, an immediate enlargement of the surgical excision was performed after consultation with the surgeon.

Follow-up protocols included unilateral mammography six months after the procedure, followed by mammographic assessment supplemented with additional imaging modalities (e.g., ultrasound, contrast–enhanced mammography or magnetic resonance imaging) as necessary based on individual clinical scenarios, such as the presence of dense breasts or a personal history of breast cancer.

Upgrade to malignancy was defined as the diagnosis of an invasive carcinoma or ductal carcinoma in situ either at surgical excision or during the follow-up period. In the latter case, malignancy had to be confirmed by biopsy or subsequent surgery. All other B3 lesions were considered as not upgraded.

### 2.4. Statistical Analysis

Continuous variables are reported as median and interquartile range (IQR), and categorical variables as absolute numbers and percentages. Upgrade rates were calculated with 95% confidence intervals (95% CIs) overall, according to B3 subtypes, and according to the complete or partial removal of microcalcifications. Comparisons of upgrade rates among different B3 subtypes were conducted with the χ^2^ and Fisher’s exact tests, as appropriate, while the Mantel–Haenszel test was used to compare upgrade rates stratified according to the complete or partial removal of microcalcifications, both overall and among B3 subtypes. All analyses were performed with STATA (version MP 18.1; StataCorp LLC, College Station, TX, USA).

## 3. Results

### 3.1. Patients and Lesion Subtypes

**Figure 1:**
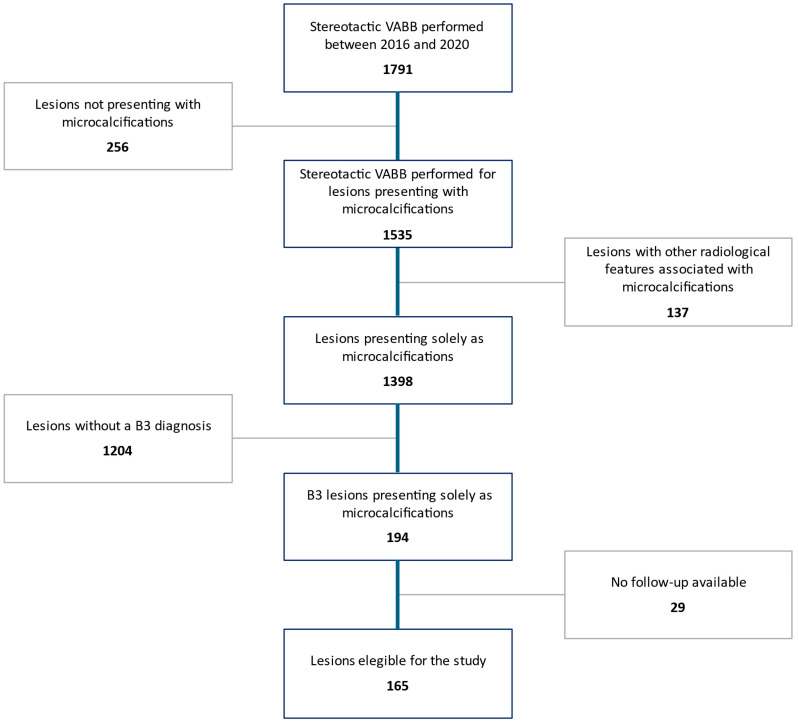
Patient selection process.

As detailed in Figure 1, out of a total of 1791 stereotactic VABB procedures recorded in our database in the inclusion timeframe, 256 cases were excluded because the target lesion was not related to microcalcifications, 137 because of the presence of other radiological features associated with microcalcifications, and 1204 because these lesions were not diagnosed as B3. Among the 194 B3 lesions presenting solely as microcalcifications, 29 were further excluded due to unavailability of follow-up data.

This resulted in a final study cohort of 165 patients (median age 54 years, IQR 47–60 years) with 165 lesions, 83/165 (50.3%) in the left breast and 82/165 in the right breast (49.7%). The overall median extension of microcalcifications was 15 mm (IQR 5–15 mm).

At biopsy, the lesions were categorized as follows: FEA was the most common subtype accounting for 53/165 lesions (32.1%), followed by ADH and LN with 40/165 cases (24.2%) each. PL were identified in 18/165 biopsies (11%) while RS were observed in 14/165 biopsies (8.5%). Following the multidisciplinary evaluation at the time of diagnosis, 74/165 patients (44.8%) underwent surgical excision, while the remaining 97/165 (58.8%) were referred to imaging follow-up.

### 3.2. Microcalcification Removal

Among the 165 lesions, complete removal was achieved in 99 cases (60.0%), while 66 lesions (40.0%) were only partially removed.

**Figure 2:**
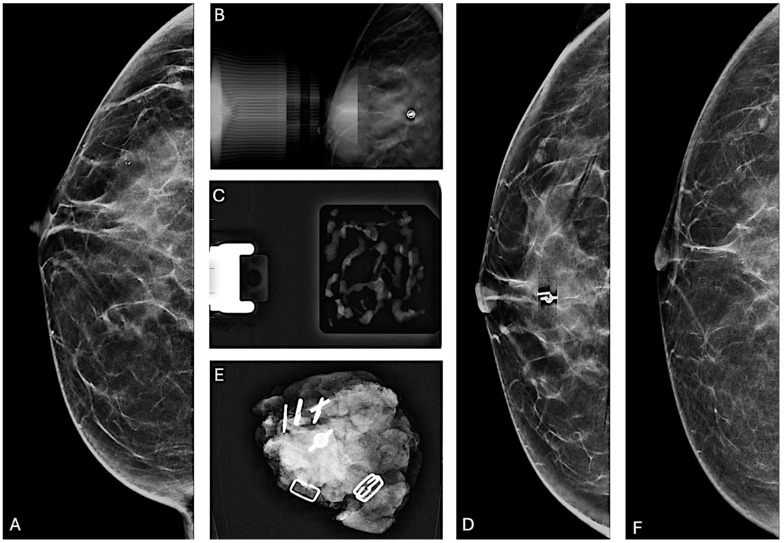
**Partial removal of microcalcifications.** (**A**) Cranio-caudal mammographic view of the right breast of a 48-year-old woman with a family history of breast cancer: a cluster of granular and punctate microcalcifications extends for 2 cm in the central quadrant, categorized as BI-RADS 4B. (**B**) Post-VABB magnification view shows partial removal of the microcalcifications with the placement of a biocompatible, non-magnetic metallic localization clip at the biopsy site. (**C**) Specimen radiograph of the biopsy cores obtained from VABB shows the presence of microcalcifications. Pathologic examination revealed the presence of FEA. After a multidisciplinary meeting, considering the family history of breast cancer and the partial removal of the microcalcifications, surgical resection was recommended. (**D**) Preoperative cranio-caudal mammographic view of the right breast with placement of a magnetic localization marker (Magseed) at the site of the localization clip. (**E**) Surgical specimen containing the Magseed, localization clip, and residual microcalcifications. Pathologic examination revealed the presence of foci of well-differentiated ductal carcinoma in situ. (**F**) Cranio-caudal mammographic view of the right breast two years after the surgical procedure, showing no suspicious mammographic findings.

**Figure 3:**
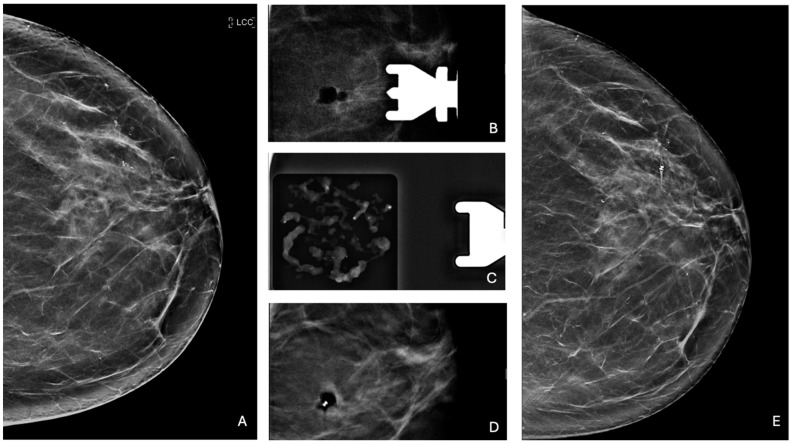
**Complete removal of microcalcifications.** (**A**) Cranio-caudal mammographic view of the left breast of a 60-year-old woman with no family history of breast cancer: a cluster of granular microcalcifications extends for 9 mm in the upper outer quadrant, categorized as BI-RADS 4B. (**B**) Post-VABB magnification view shows complete removal of the microcalcifications with a radiolucent area corresponding to the post-procedural hematoma. (**C**) Specimen radiograph of the cores obtained from VABB shows the presence of microcalcifications. Pathologic examination revealed the presence of PL. (**D**) Placement of a biocompatible non-magnetic metallic localization clip at the biopsy site. (**E**) Cranio-caudal mammographic view of the left breast at 3-year follow-up, without any new microcalcifications at the site of the previous biopsy, with the marker clip in place.

Specifically, among ADH cases, 26 out of 40 lesions (65.0%) were completely removed, while the remaining 14 (35.0%) underwent partial removal. Similarly, 23 out of 40 LN lesions (57.5%) were completely removed, whereas 17 lesions (42.5%) underwent partial removal. For FEA lesions, 33 out of 53 (62.3%) were completely removed, while 20 lesions (37.7%) were partially removed. PL showed the lowest rate of complete removal, with 6 out of 18 lesions (33.3%) fully removed, leaving 12 lesions (66.7%) partially removed. In contrast, complete removal was achieved in the majority of RS cases, with 11 out of 14 lesions (78.6%) entirely removed and only 3 (21.4%) partially removed. No statistically significant difference was observed among B3 lesions in terms of microcalcification complete or partial removal (*p* = 0.092). Examples of lesion management are presented in Figure 2 and Figure 3.

### 3.3. Upgrade to Malignancy

#### 3.3.1. Overall Upgrade Rate and Upgrade Rates Among B3 Subtypes

The overall upgrade rate to malignancy among all B3 lesions was 8.5% (14/165; 95% CI 5.1–13.7%). No statistically significant differences in upgrade rates were observed among the B3 subtypes (*p* = 0.562): ADH exhibited the highest upgrade rate at 12.5% (5/40), followed by PL at 11.1% (2/18), FEA at 9.4% (5/53), and LN at 5.0% (2/40), while no upgrades were observed among RS (0/14). In particular, we observed six ductal carcinomas in situ (three upgrades from ADH and three upgrades from FEA), seven invasive ductal carcinomas (two upgrades from ADH, two upgrades from FEA, one upgrade from LN and two upgrades from PL) and one invasive lobular carcinoma (upgrade from LN).

#### 3.3.2. Upgrade Rates According to Microcalcification Removal

The 4.0% upgrade rate (95% CI 1.6–9.9) for completely removed lesions (4/99) was significantly lower (*p* = 0.019) than the 15.2% upgrade rate (95% CI 8.4–25.7%) found among partially removed lesions (10/66), with a 0.24 odds ratio for upgrade (95% CI 0.05–0.87) among completely removed lesions compared to partially removed ones.

#### 3.3.3. Upgrade Rates of Completely and Partially Removed Lesions Among B3 Subtypes

Analysis of upgrade rates stratified according to lesion removal across B3 lesion subtypes again revealed significantly lower upgrade rates for completely removed lesions (Table 1), as confirmed by Mantel–Haenszel test (Mantel–Haenszel χ^2^ = 5.82, *p* = 0.016). For ADH, the upgrade rate was 7.7% (2/26) among completely removed lesions, compared to 21.4% (3/14) among partially removed ones (odds ratio 0.31, 95% CI 0.02–3.17). Similarly, completely removed FEA showed an upgrade rate of 3.0% (1/33), compared to 20.0% (4/20) in partially removed lesions (odds ratio 0.13, 95% CI 0.00–1.45). For PL, no upgrades were observed among completely removed lesions (upgrade rate 0.0%, 0/6; odds ratio 0, 95% CI 0–4.00) compared to the 16.7% upgrade rate (2/12) among partially removed lesions. In contrast, LN demonstrated comparable upgrade rates between the two groups, with an upgrade rate of 4.3% (1/23) among completely removed lesions (odds ratio 0.73, 95% CI 0.01–60.62) and an upgrade rate of 5.9% (1/17) among partially removed ones. Finally, RS showed no upgrades to malignancy in either group, maintaining an upgrade rate of 0.0% for both partially (0/3) and completely (0/11) removed lesions.

## 4. Discussion

In recent years, the clinical management of B3 lesions has been the subject of several guidelines, including those jointly developed by EUSOMA, EUSOBI, ESP, and ESSO [4], by the Second and Third International Consensus Conferences [6,22], and by the NHS Breast Screening Programme [23]. However, the management of these lesions remains a controversial topic [5].

As suspicious microcalcifications are one of the most common radiological patterns of presentation of B3 lesions, the large calibre needles employed in VABB enable both diagnostic precision and potential therapeutic excision. It is estimated that 3% to 11% of VABB procedures performed for microcalcifications yield a histological diagnosis of a B3 lesion [13].

A critical aspect of the management of B3 lesions lies in determining whether surgical excision or imaging follow-up is the most appropriate step after VABB. The role of the multidisciplinary meeting is pivotal, as it ensures that the management strategy is tailored to the individual needs of the patient, optimizing clinical outcomes and minimizing unnecessary interventions [24,25]. Decision-making is driven by several factors, including the radiological features, the completeness of lesion removal, the specific B3 subtype, and the associated risk of malignancy. Understanding the implications of complete versus partial lesion removal at VABB is therefore essential to optimize patient outcomes, avoid unnecessary surgeries and overtreatment of these lesions, and reduce the risk of missed malignancies [26].

Our study, conducted at a third-level cancer center, aimed to evaluate whether complete or partial removal through stereotactic VABB of B3 lesions presenting solely as microcalcifications influences the upgrade to malignancy, both overall and across different B3 subtypes. The 165 B3 lesions retrieved from our large case series of 1791 stereotactic biopsies had an 8.5% overall rate of upgrade to malignancy, slightly lower than those reported in other studies considering B3 lesions presenting solely as microcalcifications, such as the studies by Mariscotti et al. (12.7%) [27] and by Clauser et al. (13.1% in training set) [28]. This discrepancy may partially stem from differences in the reference standards: the aforementioned studies focused on lesions that underwent surgical excision, enabling the detection of subtle low-grade malignant lesions—which may not manifest overt signs of disease during follow-up and can remain indolent [29,30]—and with a potential selection bias towards B3 subtypes with higher risks of upgrade; conversely, our study covered all B3 subtypes and utilized a combination of surgical outcomes and long-term follow-up data.

In our analysis, the differences in upgrade rates across the different B3 subtypes did not reach statistical significance, which can likely be attributed to the limited number of cases within each subtype. Although no statistically significant differences in upgrade rates were observed among the B3 subtypes, the upgrade rates were consistent with those reported in the literature [31]. Notably, ADH exhibited the highest upgrade rates, aligning with its known risk profile [32,33]. Conversely, RS showed no upgrades in this cohort, which is coherent with the literature, where the reported risk of subsequent malignancy for RS remains approximately 1% [34].

Regarding the upgrade rates stratified according to the type of lesion removal, our study showed a relevant finding: lesions that were completely removed had a significantly lower upgrade rate (4.0%) compared to those that were partially removed (15.2%), with a protective 0.24 odds ratio for upgrade among completely removed lesions. The evidence that complete removal of B3 lesions during biopsy can potentially reduce the need for subsequent surgical interventions reinforces the importance of aiming for complete removal in the initial biopsy, thereby potentially avoiding overtreatment and decreasing the likelihood of developing malignancy in the long term. Our findings are consistent with previously reported ones [35,36,37,38,39] and with the very recent paper by Bianchi et al. [19], who observed a 19.6% upgrade rate among partially removed B3 lesions versus an 8.5% upgrade rate among completely removed ones, with a 0.38 odds ratio for upgrade among completely removed B3 lesions compared to partially removed ones. Our findings also align with those of the 2020 study by Mariscotti et al. [27], where wide clusters of microcalcifications (>1 cm) were associated with an increased risk of subsequent malignancy due to diagnostic underestimation at biopsy: although in that study the presence of residual microcalcifications after biopsy was not a significant predictor of upgrade to malignancy, their findings on large clusters of microcalcifications still strongly point to the fact that cases where complete removal is most difficult to achieve are those where subsequent risk of malignancy is higher.

In our subsequent analyses according to B3 subtypes, FEA demonstrated the largest difference in upgrade rates between partially removed lesions (upgrade rate 20.0%) and completely removed lesions (upgrade rate 3.0%), with a strong protective 0.13 odds ratio for upgrade among completely removed lesions. This finding is consistent with other published studies in the literature, which have emphasized that a conservative management approach may be appropriate in cases of FEA where complete removal of microcalcifications is achieved [40,41,42]. Our findings also align with the results reported by Lucioni et al. [14], who aimed to evaluate the positive predictive value of malignancy for B3 lesions and to identify predictive factors for upgrade. The authors observed a positive predictive value of 11% for FEA, which is comparable to our upgrade rate of 9.4% and supports surveillance as a viable management strategy in cases of pure FEA, especially if a complete removal of microcalcifications is achieved. In our cases of ADH and LN, the odds ratio associated with complete versus partial removal of microcalcifications had less impact on the upgrade rate compared to other subtypes, underscoring the need for heightened vigilance in managing these cases. Particularly for ADH, which demonstrated a higher upgrade rate than other B3 subtypes [8], the literature presents conflicting evidence, with some studies reporting that complete removal of microcalcifications is associated with a reduced upgrade rate [36,43,44], while other authors found no statistically significant differences [35,45,46]. For example, Schiaffino et al. [17] reported that among 65 women with ADH associated with microcalcifications that were completely removed through stereotactic VABB, only one patient developed low-grade ductal carcinoma in situ after 48 months, corresponding to a malignancy rate of less than 2%. Notably, the recent European guidelines [4] have introduced a 15 mm cutoff as a criterion for considering VAE in ADH cases. This threshold aims to optimize patient management by balancing the risks of under-treatment against the potential for overtreatment, providing a framework for more precise decision-making in these high-risk scenarios. Conflicting evidence also emerged for LN, where our study showed comparable upgrade rates among completely and partially removed lesions (5.9% and 4.3%, respectively): while the European guidelines [4] endorse the possibility of conservative management for completely removed lesions, the multicentric study by Mariscotti et al. [27]—focusing on B3 lesions presenting as microcalcifications—had identified LN as an independent risk factor for upgrade to malignancy (odds ratio 2.3, *p* = 0.03), highlighting the need for careful assessment in these cases. Finally, PL require a distinct analysis: in our study, upgrades were observed exclusively in partially removed PL. This finding is likely influenced by two factors: the relatively small number of PL included in our cohort and the characteristics of the two lesions that underwent upgrade. Both occurred in patients with extensive clusters of microcalcifications (>3 cm), a scenario associated with an increased risk of diagnostic underestimation. The absence of upgrades during follow-up in our study aligns with the existing literature, which consistently reports no upgrades in cases of PL without atypia managed with VABB [47], and further support the conservative management approach for this subtype of B3 lesions, particularly when complete removal is achieved.

Overall, the results of our study provide valuable insights to shape the management of B3 lesions presenting solely as microcalcifications, emphasizing the importance of a multidisciplinary approach integrating imaging findings with clinical and anamnestic parameters to guide the management strategy for each patient. In this context, Clauser et al. [28] proposed a diagnostic algorithm for the management of B3 lesions presenting solely as microcalcifications: their algorithm incorporates key features such as calcification morphology (linear or pleomorphic), the presence of atypia, and concomitant malignancies, highlighting the role of personalized risk assessment in determining the appropriate course of action. In our study, the difference in upgrade rates for B3 lesions according to lesion removal indicates that complete removal of the lesions can significantly reduce the risk of underestimating the presence of malignancy. Conversely, the higher upgrade rates observed among partially removed lesions highlight the need for a more cautious approach toward this type of removal, along with potentially more rigorous follow-up. This follow-up could include personalized radiological surveillance comprising not only mammography and ultrasound but also magnetic resonance imaging [48,49,50,51] and contrast-enhanced mammography [52,53,54].

It is important, however, to acknowledge certain limitations of this study. This is a retrospective study and its data reflect routine clinical practice within a third-level referral center for breast cancer care, which may have introduced selection bias and biases related to the high level of expertise of the involved clinicians, thereby limiting the generalizability of our results. The limited sample size, both overall and for certain subgroups such as PL and RS—which rarely present solely as microcalcifications—underscores the need for further studies to confirm these findings in larger multicenter cohorts. Another limitation lies in the potential interobserver variability in the interpretation of follow-up imaging studies for cases that did not undergo surgery. Regarding specific analyses, we did not evaluate if morphological features of the microcalcification clusters might have influenced the upgrade rate. Additionally, the three-year follow-up period considered in this study could be extended in future research to enhance the robustness of the obtained data.

## 5. Conclusions

This study showed how the interplay between B3 lesions subtypes and complete removal of microcalcifications influences the upgrade to malignancy of these lesions. Specifically, the complete removal of microcalcifications was associated with a lower upgrade rate in several subtypes, in particular for FEA, supporting the feasibility of conservative management in selected cases. However, for subtypes such as ADH and LN, while higher upgrade rates were still observed among partially removed lesions, the difference between the upgrade rates of complete and partially removed lesions was less pronounced, particularly in the case of LN. All these results highlight the need for a tailored approach within multidisciplinary decision-making in the management of B3 lesions, which needs to be supported by focused research to further refine risk stratification models and validate optimized treatment and follow-up algorithms.

## Figures and Tables

**Table 1:** Upgrade rates of the 165 B3 lesions included in the study.

B3 Lesion Subtype	Overall Upgrade Rate	Upgrade Rate Among Completely Removed Lesions	Upgrade Rate Among Partially Removed Lesions	Odds Ratio (95% CI)
ADH	12.5% (5/40)	7.7% (2/26)	21.4% (3/14)	0.31 (0.02–3.17)
LN	5.0% (2/40)	4.3% (1/23)	5.9% (1/17)	0.73 (0.01–60.62)
FEA	9.4% (5/53)	3.0% (1/33)	20.0% (4/20)	0.13 (0.00–1.45)
PL	11.1% (2/18)	0.0% (0/6)	16.7% (2/12)	0.00 (0.00–4.00)
RS	0.0% (0/14)	0.0% (0/3)	0.0% (0/11)	—

ADH: atypical ductal hyperplasia; LN: lobular neoplasia; FEA: flat epithelial atypia; PL: papillary lesions; RS: radial scar.

## Data Availability

The raw data supporting the conclusions of this article will be made available by the authors upon reasonable request.

## Abbreviations

VABB	Vacuum-assisted breast biopsy
BI-RADS	Breast Imaging Reporting & Data System
CI	Confidence interval
ADH	Atypical ductal hyperplasia
FEA	Flat epithelial atypia
LN	Lobular neoplasia
PL	Papillary lesion
RS	Radial scar
IQR	Interquartile range
